# Specific antigens in malignancy-associated membranous nephropathy

**DOI:** 10.3389/fmed.2024.1368457

**Published:** 2024-04-15

**Authors:** Xiaoying Hu, Guoqin Wang, Hong Cheng

**Affiliations:** Division of Nephrology, Beijing Anzhen Hospital, Capital Medical University, Beijing, China

**Keywords:** membranous nephropathy, malignant tumor, THSD7A, NELL-1, PLA2R

## Abstract

Membranous nephropathy (MN) is a glomerular disease mediated by autoimmune complex deposition, with approximately 30% of cases attributed to secondary causes. Among them, malignant tumors are a significant cause of secondary MN. Recent advancements in the identification of MN-specific antigens, such as THSD7A and NELL-1, suggest a potential association with malignant tumors, yet definitive proof of this relationship remains elusive. Therefore, this article aims to review the distribution of MN-specific antigens in patients with MN caused by malignant tumors and the possible role of these antigens in the pathogenesis of the disease.

## Introduction

Membranous nephropathy (MN) is the most common pathological type of adult nephrotic syndrome, predominantly occurring in middle-aged and elderly patients. It is caused by the deposition of immune complexes along the glomerular basement membrane (1). Approximately 30% of membranous nephropathy cases are attributed to secondary factors (2), among which malignant tumors are one of the common causes, accounting for about 5%–20% (3). As early as 1966, Lee et al. (4), proposed that there was a high coincidence between MN and malignant tumors, and it is currently considered that the presence of any of the following indicators should prompt an evaluation for malignancy-associated MN: (1) the patient’s malignant tumor and MN must occur within a similar time frame, usually within 5 years before or after the diagnosis of membranous nephropathy; (2) a remission of the malignant tumor accompanied by clinical remission of MN, while the recurrence of MN should occur when the malignant tumor recurs; (3) there is a pathophysiological connection between the two diseases, the same antigens are detected in both diseases. To date, more than a dozen MN-related antigens have been identified, of which THSD7A and NELL-1 are thought to be associated with malignancies. Subsequently, PLA2R, PCDH7, HTRA1 and FAT1 antigens were found to be positive in patients with malignancy-associated MN. However, the relationship between these antigens and malignancies is still unclear. This article will review the distribution and the mechanism of action of antigens in MN.

## THSD7A

Thrombospondin type 1 domain-containing 7A (THSD7A) is a specific antigen expressed in podocytes, identified after the discovery of PLA2R. Around 1%–3% of patients with MN are THSD7A positive (2), and this accounts for about 5%–10% of patients who do not have PLA2R antibodies (5). In recent years, different studies have shown varying positive rates for THSD7A antibodies in malignancy-associated MN (6, 7). In German and American cohorts, multiple studies indicated that the proportion of patients with THSD7A-positive MN combined with malignant tumors ranged from 9% to 30% (Table 1) (8). However, a Chinese cohort study in 2017 showed a positive rate of 2% for THSD7A antibodies in malignancy-associated MN patients (14). In studies concerning THSD7A-positive malignancy-associated MN, the associated cancers are highly diverse, including breast, lung, and digestive system cancers. Compared to those with THSD7A-positive MN without malignant tumors, patients with THSD7A-positive malignancy-associated MN are older, more often male, have lower serum albumin levels and higher titers of serum THSD7A antibodies (15), as well as more glomerular inflammatory cell infiltration in renal pathology (8). At present, most studies suggest that THSD7A-positive malignancy-associated MN are mainly composed of IgG1, IgG2 and IgG3, while THSD7A-positive kidney tissues in primary MN is mainly composed of IgG4 (5, 6, 10). However, some studies have reported that in renal tissues of patients with THSD7A-positive malignancy-associated MN, IgG4 can also be seen (16, 17). In primary THSD7A-positive MN patients, serum THSD7A antibody IgG subtypes are mainly IgG4; multiple studies have reported that there is no statistically significant difference in the level of THSD7A antibody IgG subtype between sera from THSD7A-positive MN patients with or without malignant tumors (10, 15).

**Table 1:** Frequencies of antigens in the MN in connection with tumors.

Antigens in the MN in connection with tumors	Antigen frequency in MN	Antigen frequency in MN with malignant tumors	References
THSD7A	2%	9%–30%	(8)
NELL-1	10%	10%–33%	(2, 9)
PLA2R	55%	5%	(10)
PCDH7	2%	20%	(11)
HTRA1	4%	7%	(12)
FAT1	1%	92%	(13)

In recent years, multiple studies have reported that THSD7A-positive malignancy-associated MN patients are positive for THSD7A staining in both renal and tumor tissues. Chen et al. (16) reported the expression of THSD7A antigen in renal tissue and lymph node tissue with tumor metastasis in a patient with non-small cell lung cancer, and the patient’s serum THSD7A antibody was positive. In patients with tumors such as rectal cancer, NF1-related neurofibromas and endometrial cancer combined with MN, THSD7A was also found to be positive in the kidney and tumor tissue (17, 18). In addition, one patient with MN who underwent multiple renal biopsies initially showed negative THSD7A staining for the first renal biopsy. However, after 1 year, upon the reoccurrence of the kidney disease, the THSD7A staining was positive. Two years later, the patient was diagnosed with bladder cancer, and THSD7A staining was also positive in the tumor tissue (19). Simultaneous positivity for THSD7A in renal and tumor tissue suggests that THSD7A may play an important role in pathogenic mechanism leading to MN associated with tumors. However, Liu et al. found that only one (16%) of nine patients with malignancy-associated MN had positive staining for THSD7A in their tumor tissues (20). Hara et al. (21) also reported negative staining for THSD7A in two cases of THSD7A-positive malignancy-associated MN. Therefore, tumors and MN antigens may not be always consistent. Although THSD7A-positive malignancy-associated MN patients with serum THSD7A antibody positivity are still rare (14, 20), Chen et al. (16) reported a lung cancer patient who developed MN during targeted drug therapy and whose serum THSD7A antibody was positive. When the kidney disease was alleviated, the patient’s serum THSD7A antibody turned negative, and the patient’s tumor was also under control. These results suggest that the correlation between THSD7A antibody and disease changes is related to the pathogenesis of THSD7A-positive malignancy-associated MN.

**Figure 1:**
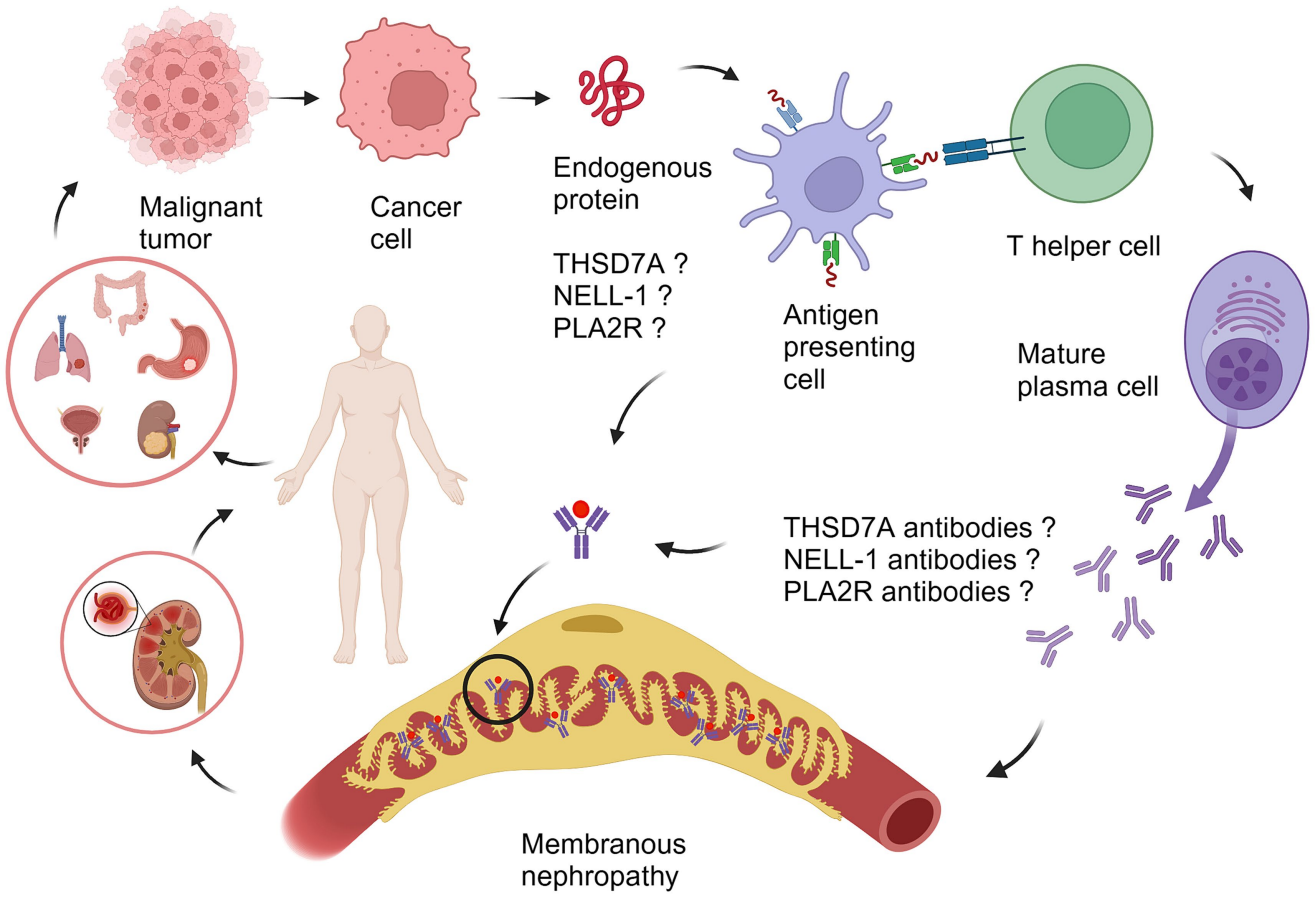
The proposed pathogenesis of malignancy-associated membranous nephropathy. Different types of tumors secrete tumor-associated antigens such as THSD7A, NELL-1, and PLA2R. The antigen is ingested by an antigen-presenting cell (APC), cut into peptide segments, presented to the APC surface, interacts with helper T cells (Th cell), specifically recognizes B cells, promotes the differentiation of B cells into mature plasma cells, secretes antibodies related to THSD7A, NELL-1, and PLA2R, and forms an antigen–antibody immune complex with the tumor antigen, which circulates to the glomerulus and deposits on the basement membrane of the glomerulus, causing MN. PLA2R, phospholipase A2 receptor; THSD7A, thrombospondin domain-containing 7A; NELL-1, neural epidermal growth factor-like 1 protein. The illustration was created with BioRender.com.

THSD7A is a transmembrane N-glycoprotein expressed in normal glomerular podocytes, endothelial cells and mesangial cells (22), while human placental vascular endothelial cells also express THSD7A (23). In recent years, THSD7A has been studied not only in MN but also in malignant tumors. Research has explored the expression of THSD7A in more than 70 types of malignant tumor tissues and found that THSD7A expresses differently in various tumors. Among them, THSD7A is relatively commonly expressed in colorectal cancer, renal cancer, breast cancer and prostate cancer (24), and it was recently found that the transcription level and protein level of THSD7A were significantly increased in gastric cancer (25). In the study of tumor pathogenesis, uncontrolled angiogenesis is considered a hallmark of malignant tumors (26), and studies have confirmed that THSD7A participates in the invasion, metastasis and generation of tumor vessels (27). *In vitro* cell culture experiments knocking out THSD7A can inhibit cancer cell proliferation and migration also confirm this mechanism (25). For MN, it is currently believed that its pathogenesis is mainly attributed to the immune system’s response to antibodies, in which B cells (plasma cells) play a major role, but T-B cells also make a significant contribution to autoimmune diseases (28). Therefore, it is speculated that THSD7A may play a role in the pathogenesis of tumor-associated MN through T cells (Figure 1). However, the mechanism of immune system abnormalities in malignant tumors is very complex considering the involvement of various genetic and environmental factors in tumor and MN onset. Therefore, there is currently no research to confirm the clear relationship between THSD7A-MN and malignant tumors. The immunological pathogenesis of THSD7A-positive malignancy-associated MN remains to be studied.

## NELL-1

Following the discovery of PLA2R and THSD7A, Sethi et al. (29) found neural epidermal growth factor-like 1 protein (NELL-1) through laser microdissection and mass spectrometry analysis of renal biopsy tissues from patients with MN who were negative for PLA2R, accounting for approximately 10% of cases of MN (2). At the same time, in this cohort, 11.7% of NELL-1 positive MN were combined with malignant tumors. Subsequently, Caza et al. (9) reported that among patients with NELL-1 positive MN in the cohort, the proportion of those combined with malignant tumors was as high as 30%, suggesting that NELL-1 is a target antigen for MN associated with malignant tumors. In our center’s cohort of 832 patients with membranous nephropathy and Japan’s cohort of 104 patients, NELL-1 positive MN patients did not combine with tumors (30). The reason for large differences between the cohorts, which may be related to geography and ethnicity. Compared with MN patients without malignant tumors (9), NELL-1 positive malignancy-associated MN patients were clinically characterized by older age and more males. No significant difference of serum creatinine and 24-h urinary protein quantification levels was found between these patients and those PLA2R-MN and THSD7A-MN patients (9). It has been reported that there was a significant increase in the number of inflammatory cells infiltrating glomeruli in malignancy-associated MN patients (31), but this feature was not observed in NELL-1 positive malignancy-associated MN patients. NELL-1 positive malignancy-associated MN glomerular capillary loop immune complex shows segmental deposition or combined mesangial region immune complex deposition, renal tissue IgG subclass mainly by IgG1, pathological performance similar to NELL-1 positive MN without combination of malignant tumors (9).

NELL-1 is a 140 kDa modular glycoprotein normally expressed in neural tissues with lower expression levels in non-neural tissues such as the kidney and liver (31, 32). NELL-1 has higher expression in renal tubules than glomeruli (33). NELL-1 has been studied in numerous types of tumors prior to the discovery of NELL-1 as a MN-specific antigen. Expression levels of NELL-1 vary across tumor types. It has been reported that NELL-1 was overexpressed at both transcriptional and protein levels in neuroblastoma and osteosarcoma (34, 35), as well as prostate cancer, lung cancer and breast cancer (36). However, NELL-1 was downregulated at the gene, transcript and protein levels in renal cancer, gastric cancer and lymphoma (37–39). In previous studies on the pathogenesis of malignant tumors, abnormal CpG island and promoter methylation of genes were closely related to the occurrence of tumors. Studies on the pathogenic mechanism of NELL-1 in malignant tumors show that abnormal CpG islands and promoter methylation lead to downregulation of NELL-1 expression, thereby regulating the malignant behavior of renal cancer cells (39). In addition to renal cancer, NELL-1 gene promoter methylation can also be detected in colorectal cancer (40), esophageal cancer (41). Therefore, NELL-1 may participate in the cell growth, differentiation and tumorigenesis of different tumors. However, despite the high expression of NELL-1 found in different malignancies, only a few patients with malignancies will continue to develop into MN. In recent years, studies on NELL-1 positive malignancy-associated MN have found that the expression of NELL-1 was observed in tumor and kidney specimens from patients with breast cancer, lymphoid follicle carcinoma and esophageal cancer (9, 42), but no study has confirmed a clear mechanism of action for NELL-1 in MN associated with malignant tumors. Although NELL-1 has been proven to play a role in tumorigenesis and development, its pathophysiological mechanism in malignant tumors remains to be further studied.

## PLA2R

It is widely acknowledged that PLA2R is the most important antigen in primary membranous nephropathy, but a study has revealed that 16 (5.3%) of the 302 patients with PLA2R antibody-positive MN were found to have malignant tumors within 24 months after the diagnosis of MN (10). There are also reports show that 3 (25%) of the 12 patients with malignancy-associated MN were positive for PLA2R, suggesting that PLA2R may be associated with malignancy-associated MN. However, in the study conducted by Uhlen M, no remission of MN was observed after anti-tumor treatment in patients with PLA2R-positive MN combined with malignant tumors. It is considered that the relationship between PLA2R-positive MN and malignant tumors may be a coincidence (43). Therefore, the relationship between PLA2R and malignancy-associated MN needs further clarification. Compared with PLA2R-positive MN without malignancy, patients with PLA2R-positive malignancy-associated MN were older, had more proteinuria and worse renal function, and showed heavier interstitial fibrosis pathologically (10). Zhao et al. found that IgG subclass IgG3 was mainly positive (14) in renal tissues of PLA2R-positive malignancy-associated MN, while it was mainly positive for IgG4 (44) in primary MN with PLA2R positivity. It is considered that negativity of PLA2R and IgG4 in renal tissue are indicators of malignancy-associated MN (45, 46). In patients with PLA2R-positive malignancy-associated MN, serum PLA2R antibody IgG subtypes were found to be consistent with those of primary MN (10, 46), suggesting that the pathogenesis of PLA2R-positive malignancy-associated MN and PLA2R-positive primary MN may have pathways in common.

PLA2R is a type I transmembrane receptor glycoprotein expressed on the surface of glomerular podocytes (44). PLA2R expression has been found in both kidney and malignant tumor tissues, but the role of PLA2R positivity in the pathogenesis of malignancy-associated MN remains unclear. In recent years, the research on PLA2R in tumors has gradually increased. Through the analysis of tumor gene chip database, it was found that the expression level of PLA2R varied in different tumor tissues, and the expression level of PLA2R mRNA was decreased in breast cancer (47) and kidney cancer (48), but not in pancreatic cancer and gastric cancer (47). In addition, there are controversies about the role of PLA2R in tumor cells. Some studies have found that PLA2R can promote apoptosis and inhibit cell transformation in tumor tissues (47, 49, 50). Vindrieux et al. (48) found that knockdown of PLA2R promotes the formation of cancer cell colonies in renal cancer cell lines. In addition, the death of cancer cells was observed in PLA2R-expressing cancer cells due to an increase in intracellular reactive oxygen species (ROS), suggesting that PLA2R promotes tumor cell death. However, research by Jones et al. found that both renal cancer (48) and breast cancer tissues were observed to have PLA2R promoter methylation phenomenon. The methylation of PLA2R promoter would inhibit the expression of tumor suppressor genes in cancer cells, thus promoting cancer cell proliferation (51). Since there are still few case reports of PLA2R-positive MN patients with tumors in which PLA2R is expressed simultaneously in tumor tissues, the role of PLA2R in the pathogenesis of kidney and malignant tumors is not clear, which warrants further research.

## PCDH7, HTRA1, FAT1

In addition to the MN specific antigens mentioned above, several newly discovered MN antigens may also be associated with malignant tumors. Approximately 20% of patients with Protocadherin 7 (PCDH7)-MN have malignant tumors (11). Previous studies have shown that PCDH7 is involved in tumorigenesis in bladder cancer, renal cancer, lung cancer and gastric cancer (52–57), but its pathogenesis in MN remains unclear. Approximately 7% of patients with high-temperature requirement A1 (HTRA1)-MN have concomitant malignant tumors (12). Although HTRA1 has not been fully studied in the kidney, previous studies on malignant tumors suggest that HTRA1 has tumor suppressive effects (58–60). The loss of expression of HTRA1 leads to an increase in tumor invasiveness, enhanced metastatic ability and chemotherapeutic resistance (60). Protocadherin FAT1 (FAT1) is associated with hematopoietic stem cell transplant (HSCT)-related MN (0.6%) (13). As one of the most common mutant genes in a variety of cancers (61), whether FAT1 has a clear mechanism of action in HSCT-related MN requires further investigation.

It is necessary to acknowledge the limitations of this review. For example, we did not delve into the specific pathogenic mechanisms of antigens associated with malignancies in MN, as there is currently a lack of research on the pathogenic mechanisms in these patients. The second restraint is the limited number of cases of tumor-associated MN, especially those with immunostaining of antigens in both renal and tumor tissues. Therefore, further understanding of the characteristics of antigens in tumor-associated MN requires studies with larger sample sizes and longer follow-up time.

## Conclusion

In this article, we summarize the clinical and pathological characteristics of antigen-positive malignancy-associated MN reported in recent years. We also summarize the possible role of MN-specific antigens in the pathogenesis of malignancy-associated MN. Due to the complexity of the pathogenesis of malignant tumors, further explorations of the role of antigens in the pathogenesis will provide therapeutic targets for diseases in the future.

## Author contributions

XH: Conceptualization, Data curation, Methodology, Software, Writing – original draft, Writing – review & editing. GW: Project administration, Supervision, Validation, Visualization, Writing – review & editing. HC: Funding acquisition, Supervision, Validation, Visualization, Writing – review & editing.
